# Age Is the Only Predictor of Poor Bowel Preparation in the Hospitalized Patient

**DOI:** 10.1155/2016/2139264

**Published:** 2016-04-20

**Authors:** Julia McNabb-Baltar, Alastair Dorreen, Hisham Al Dhahab, Michael Fein, Xin Xiong, Mike O' Byrne, Imene Ait, Myriam Martel, Alan N. Barkun

**Affiliations:** ^1^Division of Gastroenterology, Hepatology and Endoscopy, Brigham and Women's Hospital, Harvard Medical School, Boston, MA, USA; ^2^Division of Gastroenterology, McGill University, Montreal, QC, Canada; ^3^Royal Hospital, Muscat, Oman

## Abstract

We examine the impact of key variables on the likelihood of inpatient poor bowel preparation for colonoscopy. Records of inpatients that underwent colonoscopy at our institution between January 2010 and December 2011 were retrospectively extracted. Univariable and multivariable logistic regression models were fitted to assess the effect of clinical variables on the odds of poor preparation. Tested predictors included age; gender; use of narcotics; heavy medication burden; comorbidities; history of previous abdominal surgery; neurological disorder; product used for bowel preparation, whether or not the bowel regimen was given as split or standard dose; and time of endoscopy. Overall, 244 patients were assessed including 83 (34.0%, 95% CI: 28.1–39.9%) with poor bowel preparation. Cecal intubation was achieved in 81.1% of patients (95% CI: 76.2–86.0%). When stratified by quality of bowel preparation, cecal intubation was achieved in only 65.9% (95% CI: 60.0–71.9%) of patients with poor bowel preparation and 89.9% (95% CI: 86.1–93.7%) of patient with good bowel preparation. In multivariate logistic regression analysis, only advancing age was an independent predictor of poor bowel preparation (OR = 1.026, CI: 1.006 to 1.045, and *p* = 0.008). Age is the only independent predictor of poor bowel preparation amongst hospitalized patients.

## 1. Introduction

The Multisociety Task Force on Colorectal Cancer recommends a target of 90% colonoscopy completion rate [1, 2], which is essential to meet the diagnostic and therapeutic aims of the procedure. However, the success of a colonoscopy relies on many factors, which include age, comorbidities, location of procedure, bowel preparation [3], and timing of endoscopy [4].

On the matter of bowel preparation, investigators have also examined characteristics associated with an adequate bowel preparation. These include admission status, gender, age, obesity, socioeconomic status, comorbidities, insurance status, number and type of medications, and time of endoscopy, as well as time between preparation and endoscopy [4–12]. Few investigators have assessed these outcomes in the context of hospitalized patients, which represents a higher risk population, where success of the procedure is likely to be more significant than the typical screening colonoscopy performed in the elective setting. Given the limited resources available and the cost of inadequate bowel preparation [13], we examine the impact of patient characteristics on the likelihood of poor bowel preparation at inpatient colonoscopy.

## 2. Methods

### 2.1. Study Design, Setting, and Participants

The records of inpatients who underwent colonoscopy at our institution between January 1, 2010, and December 31, 2011, were retrospectively extracted from EndoWorks (Olympus, Center Valley, PA). Endoscopists at our institution have a mean annual caseload of 533 colonoscopies between 2013 and 2015. Fellows and residents participated in 59% of cases, all of which were supervised. Six individuals reviewed hospital charts, and 10% of cases were audited for validation. All data collection and analyses were undertaken following the approval and institutional oversight of the Institutional Review Board for the Protection of Human Subjects.

### 2.2. Variables

The primary endpoint examined was poor bowel preparation. Patient and demographic information collected included age, gender, race, body mass index, comorbidities (cirrhosis, malignancy, renal impairment, and neurologic disorder), indication for colonoscopy, number of previous colonoscopies, previous abdominal surgery, and time between bowel preparation and start of colonoscopy, as well as time of the day and context (elective, emergent, and urgent) of endoscopy. Baseline comorbidities were determined using the Charlson Comorbidity Index (CCI) and stratified according to four levels: 0, 1, 2, and ≥3 [14, 15].

Bowel preparation was categorized according to four groups: poor, fair, good, and excellent preparation by the endoscopist. Poor bowel preparation was defined as poor or fair bowel preparation.

### 2.3. Study Size

The sample size of 500 was chosen to reflect the estimated number of events for the least frequently reported outcome of interest, for example, 23% incomplete bowel preparation, and the number of variables to be tested [16].

### 2.4. Statistical Methods

The incidence of poor bowel preparation was assessed. In univariate analyses, quantitative variables were examined as both continuous and categorized variables. The minimum *p* value approach, as described by Mazumdar and Glassman [17], was used to identify the most significant cut-offs for prediction of the outcome of interest. The most informative format of the variable was then retained for multivariable analyses. Multivariable logistic regression models for prediction of poor bowel preparation were fitted.

To assess multicollinearity between variables and CCI, we assessed the variance inflation factor (VIF) collinearity statistic and the tolerance [18]. Analyses were performed using SPSS (v21.0).

## 3. Results

Overall, 244 charts of patients undergoing colonoscopy during hospitalization were assessed. Of those, 83 (34.0%, 95% CI: 28.1–39.9%) patients had poor bowel preparation. During endoscopic examination, the cecum was reached in 198 patients (81.1%, 95% CI: 76.2–86.0%). The mean age of the patients was 66 years; 133 were men (54.5%). The rate of complete colonoscopy in patients with poor bowel preparation was 65.9% (95% CI: 60.0–71.9%) compared to 89.9% (95% CI: 86.1–93.7%) in patients with good bowel preparation (*p* < 0.001). Incomplete colonoscopy due to poor bowel preparation added a total of 64 extra days of admission before performing repeat colonoscopy or CT-colonography, in 21 patients, representing a mean additional hospitalization duration of 3 days.

Overall, 40.2% of patients had previous abdominal surgery, 31.4% were diabetic, 29.5% had limited mobility, and 10.3% had a neurologic condition. The comorbid score was greater than or equal to 3 in half the patients. Medication history was also assessed; overall 37.4% of patients received more than 8 medications and 17.4% were on narcotics.

In univariable analyses, the only clinical variable associated with a poor bowel preparation was advancing age. The median age of the patients with poor bowel preparation was 76 versus 67 in patients with adequate preparation (*p* < 0.001). Certain factors such as diabetes (*p* = 0.06), limited mobility (*p* = 0.075), and elevated comorbid score (CCI ≥ 3, *p* = 0.068) trended towards significance (Table 1). Bowel preparation was most often achieved using a PEG solution preparation (88.9%). Split preparation was only used in 9.9% of patients.

In multivariable logistic regression analyses, adjusting for gender, CCI, diabetes, and poor mobility, age remained independently and significantly predictive (OR = 1.026, CI: 1.006 to 1.045, and *p* = 0.008). The model obtained a VIF of 2.52 and a tolerance of 0.40. Based on this, we calculated that, for every 10-year increase in age, the likelihood of poor bowel preparation increased by 1.29 (Table 2).

The predetermined sample size of 500 was not achieved during the study because of limitation in the study period.

## 4. Discussion

Colonoscopy is an important diagnostic and therapeutic tool to assess and treat patients hospitalized with gastrointestinal disease. Adequate colonic mucosa visualization is necessary for proper assessment and is related to the quality of bowel preparation [19–21]. Hospitalization status has been identified as a risk factor for poor bowel preparation, but the reason for this association is unclear [5, 6]. Our study is the first to focus on predictors of poor bowel preparation specifically in hospitalized patients.

In this cohort, the rate of poor bowel preparation was 34.0% (95% CI: 28.1–39.9%), which is similar to previously published data where rates range from 17 to 38% [6, 8, 9, 11, 12, 16, 22–24]. The rate of complete colonoscopy in patients with poor bowel preparation was 65.9% (95% CI: 60.0–71.9%). Overall, the rate of cecal intubation was 81.1% (95% CI: 76.2–86.0%), which is comparable to previously published data in hospitalized patients, reporting a cecal intubation rate of 88% (95% CI: 79–97%) [25].

We identified that age was a key factor predicting poor bowel preparation in hospitalized patients. In uni- and multivariable analysis, age was the only significant predictor of poor preparation. Furthermore, for every 10-year increase in age, the odds of having poor bowel preparation increased by 1.29. Age has been previously described as a predictor of poor bowel preparation [3, 5, 8, 11], but this is the first such report specifically in a hospitalized patient population.

The high rate of poor bowel preparation and subsequent lower cecal intubation rate may have been impacted by our cohort's comorbid status. Moreover, increasing comorbidities has been identified previously as causally related to inadequate bowel preparation in primary outpatient populations [6, 8, 9, 11]. In our study, diabetes and elevated comorbid score trended towards statistical significance as predictors of inadequate preparation. Diabetes may contribute to poor bowel preparation because of associated autonomic neuropathy and subsequent constipation. Our study was underpowered and thus assessing a greater number of patients would be necessary to confirm that these factors are indeed significant predictors of poor bowel preparation.

Inpatient status has been identified as a predictor of poor bowel preparation [5, 23]. Even with adequate preparation, completion rates of colonoscopies in inpatients have been documented to be lower than those of outpatients [23]. Comparative data from 1988 and 2008 have suggested that inpatient colonoscopies are currently being performed on markedly sicker patients [25]. Interestingly, cecal intubation rates have increased over time, owing largely to improved endoscopic techniques and technology [25]. It is unclear what the driving force behind inadequate bowel preparation is; however, it is likely that based on the available observational data in sicker patients adequate bowel preparation is harder to achieve.

Patients with poor bowel preparation stayed an average of 3 additional days in hospital. This raises concern of increased costs to the health care system. A study from 2002 documented that incomplete examinations were associated with a 12% and 22% increase in costs in private and public hospitals, respectively [13]. This highlights a significant burden to the health care systems, suggesting that measures should be taken to increase successful preparation. Although the observational and retrospective nature of our study shows an association (and does not ascertain for cause), one may advocate for an age-specific approach to inpatient bowel preparation, with attention given to elderly patients who may have difficulty completing their preparations, to avoid potentially unnecessary health care expenditures due to poor preparation among elderly hospitalized individuals.

The limitations of our study include its retrospective design, which can lead to bias, as well as being underpowered. It is possible that we did not observe any significant differences because of the inability to achieve the desired sample size. The lack of power may explain why other patient-centered factors, particularly comorbid status, were not significant in our data set. Preparation-based factors such as split-dose preparation and runway time could also have been affected by the lack of power thus leading to a type 2 error. Other possible reasons for not duplicating findings from other studies would include variations in patient characteristics, including language barriers, as well as nursing and medical practices. Moreover, bowel preparation was not standardized at our institution, which may represent an important confounder. On that matter, there are potentially other unmeasured confounders that may ascertain for our findings. For example, sicker patients were potentially unable to take the prep and had a longer hospital stay due to their multimorbidity.

In conclusion, 34.0% of inpatients had poor bowel preparation and the rate of complete colonoscopy in this group was 65.9% (95% CI: 60.0–71.9%). In uni- and multivariable analysis, age was the only independent significant predictor of poor bowel preparation. An age-specific approach should be implemented to minimize this factor in relation to poor bowel preparation. The broader use of split-dosing preparations may result in increased bowel cleanliness in this patient population, although high quality evidence in this difficult group is lacking at present.

## Additional Points

At the authors' tertiary care referral center, poor bowel preparation is frequently reported (34.0%) in the hospitalized settings. In the hospitalized patients, age is the only independent predictor of poor bowel preparation.

## Figures and Tables

**Table 1:** Characteristics of patients as a function of bowel preparation.

	Overall	Poor bowel preparation	Adequate bowel preparation	*p* ^*∗*^
*Number of patients (%)*	244	85 (34.8)	158 (64.8)	
Median age [IQR]	71 [58, 79]	76 [64.5, 82]	67 [53, 77]	<0.001^t^
*Gender (%)*				
Male	54.7	52.9	55.7	0.688
Female	45.3	47.1	44.3	
*Comorbidity (%)*				
Diabetes	31.4	39.3	27.2	0.06
Neurologic condition	10.3	14.6	6.5	0.296
Limited mobility	29.5	36.9	25.5	0.075
Previous abdominal surgery	40.2	34.6	43.2	0.253
*Charlson Comorbidity Index (CCI) (%)*				
0	20.2	16.7	22.2	0.068
1	12.8	8.3	15.2	
2	16.5	13.1	18.4	
≥3	50.4	61.9	44.3	
*Medication (Rx) (%)*				
More than 8 Rx	37.4	42.4	35.0	0.270
Narcotic	17.4	21.4	15.3	0.285
*Bowel preparation and procedure (%)*				
PEG with electrolytes	88.9	87.7	89.5	0.466
Sodium picosulfate	9.0	9.9	8.5	
Split dose	9.9	11.2	9.2	0.648
Time of procedure (PM)	58.9	58.4	59.2	0.915

^*∗*^Chi-square association between patient characteristics and bowel preparation.

^t^Mann-Whitney test performed.

**Table 2:** Multivariable analysis predicting poor bowel preparation.

	OR (95% CI)	*p* ^*∗*^
Age	1.03 (1.01–1.05)	0.016
*Gender*		
Male	Ref.	
Female	1.34 (0.76–2.36)	0.310
*CCI*		
0	Ref.	
1	0.46 (0.15–1.43)	0.177
2	0.57 (0.20–1.62)	0.288
≥3	1.16 (0.48–2.82)	0.738
*Comorbidity*		
Diabetes	1.24 (0.65–2.36)	0.520
Poor mobility	1.38 (0.74–2.56)	0.312

^*∗*^Multivariate logistic regression.
